# Medical Qualifications

**Published:** 1900-09-08

**Authors:** 


### Medical Qualifications.

The following is a list of the examining bodies, and the degrees and diplomas given by them which admit to the Register. It must be understood, however, that the Universities grant many other degrees and the Colleges many other diplomas. These, however, are of the nature of higher qualifications, and to gain them the lower ones must first be obtained. The following, then, are all that are of interest to the commencing student:—

### ENGLAND.

### The University of Oxford.

The degrees of M.B. and B.Ch., which, however, can only be obtained after taking a degree in arts.

### The University of Cambridge.

The degrees of M.B. and B.C. These may be taken without obtaining a degree in arts.

Information may be obtained on application to the Registrar of the University, the Registrary, Cambridge.

In both the above universities residence is required, and thus the fees for the examination can hardly be considered apart from the other necessary expenses.

### The University of London.

The degree of M.B., which is of itself a complete qualification. The fees come to £15, in addition to which there is a fee of £2 for matriculation, which, however, takes the place of the necessary preliminary examination in arts.

Information may be obtained on application to the Registrar of the University of London, Burlington Gardens, London, W.

### The University of Durham.

The degree of M.B., which is open to women as well as men. One year at least of the necessary five years' course must be spent in attendance at the College of Medicine, Newcastle-upon-Tyne, and it is essential that the preliminary arts examination for degrees should be passed. Including the fee for the latter the total fees for the qualifying degree amount to £26.

Information may be obtained on application to the secretary of the University of Durham College of Medicine, Newcastle-on-Tyne, or see the Calendar.

### The Victoria University.

The degrees of M.B., Ch.B. Two years at least out of the necessary five years' course must be spent at one or other of the colleges of the University, namely, Owens College, Manchester; University College, Liverpool; or the Yorkshire College, Leeds, and one of these years must be subsequent to passing the first M.B. examination. An entrance examination must be passed. Including the fees for this, the total fees for the qualifying degree amount to £17.

Information may be obtained on application to the Registrar or the Dean of either of the colleges as above, or from the Calendar.

### The University of Birmingham.

The degrees of M.B., B.Ch. In order to obtain either of these degrees it is necessary that a student shall have passed at least the first four years of his curriculum in attendance upon classes of the University. The fees, including that for matriculation, amount to £21.

### The Conjoint Examining Board in England.

This Board is formed by the Royal College of Physicians of London and the Royal College of Surgeons of England, and on passing its examinations the candidate obtains a diploma from each body, and thus becomes L.R.C.P., M.R.C.S.

The total fees for qualification amount to 40 guineas (£42), in addition to the fees for the preliminary examination in arts.

Information may be obtained from the Secretary, Examination Hall, Victoria Embankment, London, W.C.

### The Society of Apothecaries of London.

This body grants a full qualification, the L.S.A., the total fees for which amount to 15 guineas (£15 15s.), in addition to the fee for the preliminary examination in arts.

Information may be obtained on application to the Secretary to the Examiners, Apothecaries' Hall, Blackfriars, London, E.C.

### SCOTLAND

THE UNIVERSITY OF EDINBURGH.

The degrees of M.B., Ch.B. Two of the five years' course must be taken in Edinburgh. Women students are recognised if the regulations laid down for the conduct of their classes are conformed with. Including the fee for the preliminary examination, which is half a guinea, the total fees for the qualifying degrees amount to 22½ guineas. The examinations of several other bodies are accepted as equivalent to the preliminary.

Information may be obtained by application to the Dean of the Faculty of Medicine, University of Edinburgh; or from the Calendar.

THE UNIVERSITY OF GLASGOW.

The degrees of M.B., Ch.B. Residence for two years is required as at Edinburgh. The total fees for qualification are the same as at Edinburgh—namely, 22½ guineas. Women students are recognised, and the Lady Margaret College is connected with the University.

For further information see Calendar.

THE UNIVERSITY OF ABERDEEN.

The degrees of M.B., Ch.B. Two years of the necessary five years' course must be passed in Aberdeen. The total fees for the qualifying degrees amount to 22½ guineas, including the entrance examination.

For further information see Calendar.

THE UNIVERSITY OF ST. ANDREWS.

Two of the five years' course must be taken out at St. Andrews or at the University College, Dundee, and the rest at some recognised medical school. The fees for the qualifying degrees (M.B., Ch.B.) are the same as at Edinburgh.

Information may be obtained on application to the Dean of the Medical Faculty, University College, Dundee; or see Calendar.

THE SCOTTISH COLLEGES.

The Royal College of Physicians of Edinburgh, the Royal College of Surgeons of Edinburgh, and the Faculty of Physicians and Surgeons of Glasgow have instituted a system of conjoint examinations, on passing which the candidate obtains the diplomas of each body, and becomes L.R.C.P.Edin., L.R.C.S.Edin., and L.F.P.S.Glasg.

The total fees for this qualification amount to £30, including the preliminary.

Information may be obtained on application to Jas. Robertson, Esq., 48, George Square, Edinburgh; or to A. Duncan, Esq., 242, St. Vincent's Street, Glasgow.

### IRELAND.

THE UNIVERSITY OF DUBLIN.

The degrees of M.B., B.Ch., and B.A.O. These degrees are only conferred on candidates who are graduates in arts, but the arts course may be carried on concurrently with the medical course. The fees for the qualifying medical degrees amount to £17, with 5s. more for matriculation, but the fees for the arts degree must also be taken account of.

Residence is essential.

Information may be obtained on application to the Registrar of the School of Physic in the University of Dublin.

THE ROYAL UNIVERSITY OF IRELAND.

The degrees of M.B., B.Ch. These are open to both sexes. The total fees, including matriculation, &c., for the qualifying degrees amount to £17.

THE CONJOINT EXAMINING BOARD IN IRELAND.

The Royal College of Physicians of Ireland and the Royal College of Surgeons in Ireland have arranged a series of examinations, on passing which the candidate obtains the diploma of each body, and becomes L.R.C.P.I., L.R.C.S.I.

The total fees for this qualification amount to 42 guineas (£44 2s.), including the fee for the preliminary examination.

Information may be obtained from the Secretary of the Committee of Management, Conjoint Examining Board, Royal College of Physicians, 6, Kildare Street, Dublin.

THE APOTHECARIES' HALL OF IRELAND

is entitled to grant a full qualification, the fees for which amount to 21 guineas.

Information may be obtained from the Secretary of the Apothecaries' Hall, Dublin.

